# Supplementary material for: A Phytophthora receptor-like kinase regulates oospore development and can activate pattern-triggered plant immunity
Source: Nat Commun. 2023 Jul 31;14:4593. doi: 10.1038/s41467-023-40171-7 (PMC10390575; doi:10.1038/s41467-023-40171-7)
Supplement: Supplementary file 1 — Supplementary Information [file 41467_2023_40171_MOESM1_ESM.pdf]

Supplementary Information for

A *Phytophthora* receptor-like kinase regulates oospore development and can activate pattern-triggered plant immunity

This PDF file includes:

Supplementary Figures.

Supplementary Table 1.

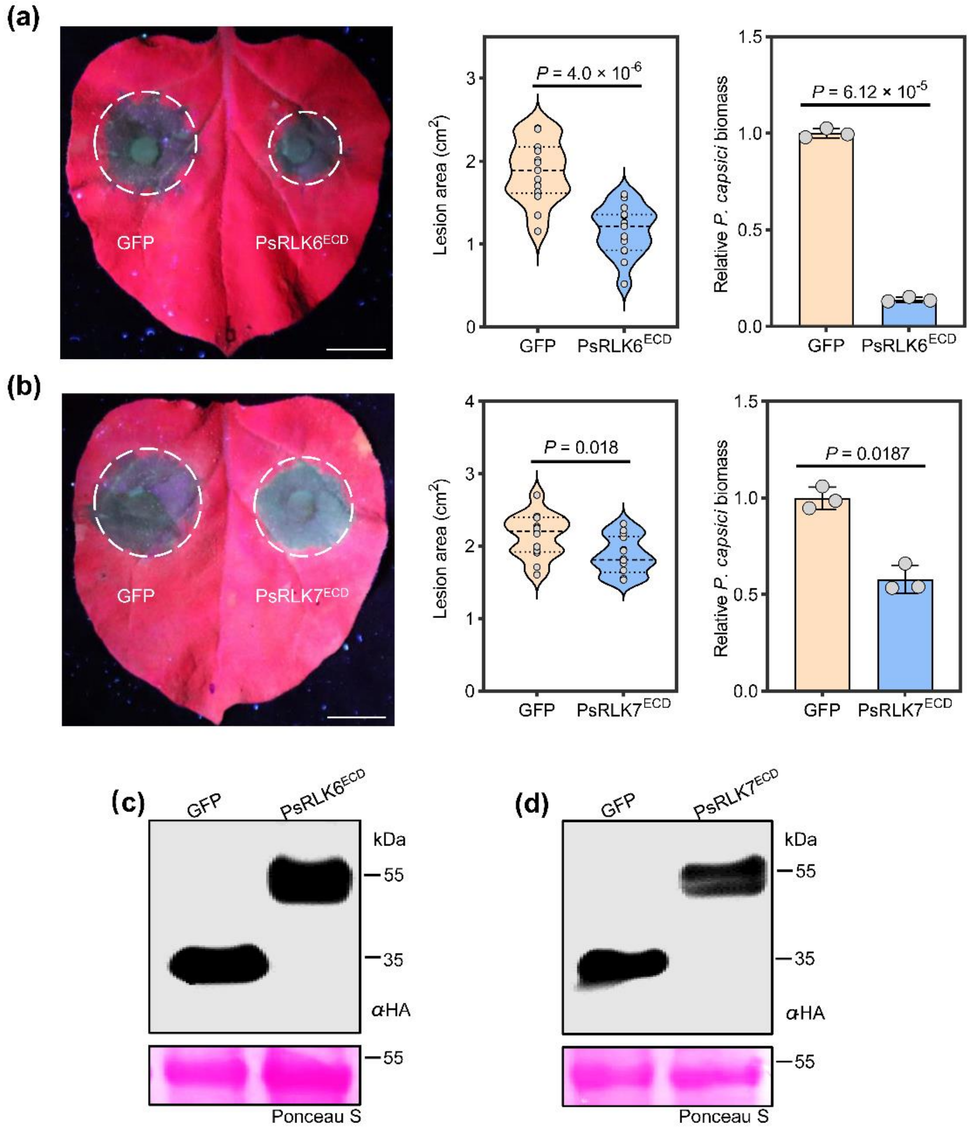

Supplementary Fig. 1. Expression of PsRLK6^{ECD} and PsRLK7^{ECD} could suppress *Phytophthora* infection. (a and b) Transient expression of PsRLK6^{ECD}-HA (a) and PsRLK7^{ECD}-HA (b) in *Nicotiana benthamiana* enhanced resistance to *P. capsici*. PsRLK6^{ECD}-HA and GFP-HA or PsRLK7^{ECD}-HA and GFP-HA were expressed in half leaves of *N. benthamiana* by *Agrobacterium*-mediated transient expression for 24h, respectively. The leaves were inoculated by *P. capsici* LT263 and photographed under UV light 36 hpi. The lesion sizes were measured, and date are shown as violin plots with individual data points plotted (n = 15 or 14 biologically independent samples). The centre line, edges and whiskers indicate the median, lower and upper quartiles and the minimum and maximum, respectively. The data of biomass were shown as mean ± SD (n = 3 biologically independent experiments). The statistical analyses were performed with two-tailed Student's t-test. Scale bars, 1 cm. The Immunoblotting showed the expression of GFP-HA, PsRLK6^{ECD}-HA (c) and PsRLK7^{ECD}-HA (d). Source data are provided as a Source Data file.

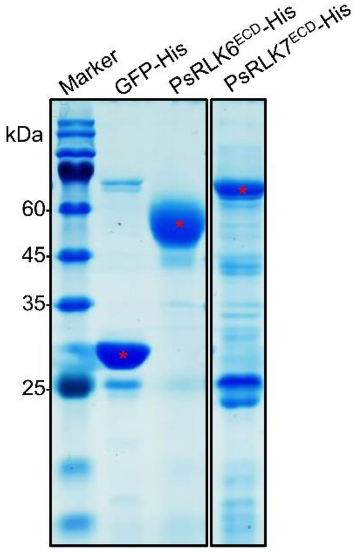

Supplementary Fig. 2. Purified GFP, PsRLK6^{ECD} and PsRLK7^{ECD} recombinant protein produced by *P. pastoris*. Coomassie brilliant blue stained the sodium dodecyl sulfate-polyacrylamide gel electrophoresis (SDS-PAGE) of GFP, PsRLK6^{ECD} and PsRLK7^{ECD} proteins. The red asterisks indicate the destination protein.

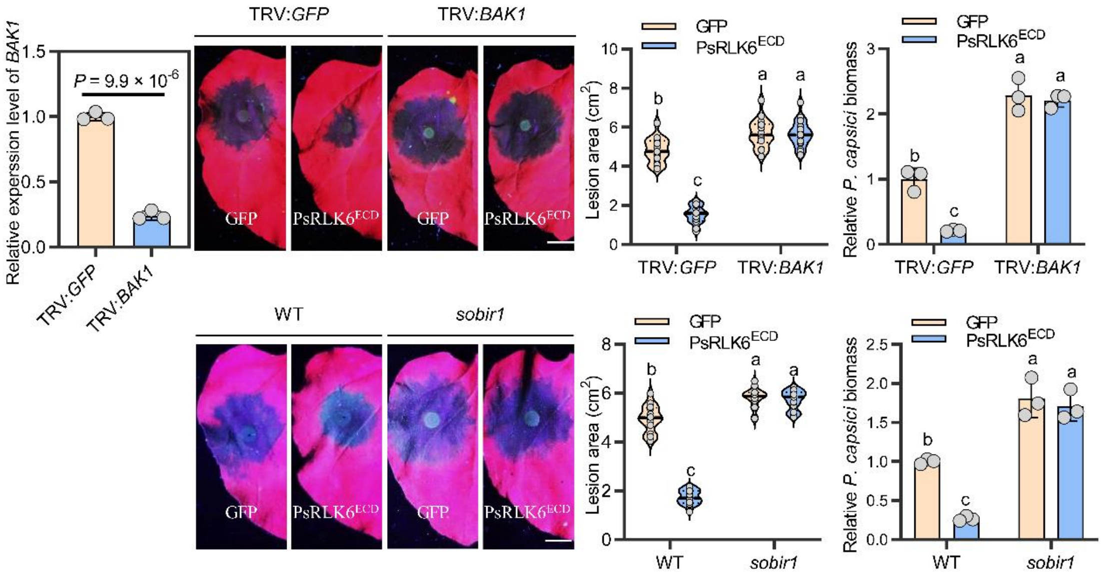

Supplementary Fig. 3. PsRLK6^{ECD}-induced disease resistance to *P. capsici* is dependent on BAK1 and SOBIR1 in *N. benthamiana*. (a) Silencing efficiency of *BAK1* in *N. benthamiana*. The transcript levels were determined by qRT-PCR, normalized with *EF-1α*, relative to TRV:*GFP*-treated leaves, which was set as 1. The data were shown as mean ± SD (n = 3). *P* value was derived by two-tailed Student's t-test. (b) Representative leaves showing the disease symptoms of *BAK1*-silencing (c) and *sobir1* (e) mutant treated by GFP *or* PsRLK6^{ECD} protein in *N. benthamiana* leaves upon infection with *P. capsici.* Lesion areas (c and f) and biomass of *P. capsici* infection (d and g) with different treatments were measured. The date of lesion areas are shown as violin plots with individual data points plotted (n = 16 - 18 biologically independent samples). The centre line, edges and whiskers indicate the median, lower and upper quartiles and the minimum and maximum, respectively. The experiment was performed three times with similar results. The data of biomass were shown as mean ± SD (n = 3 biologically independent experiments). The statistical analyses were performed with two-way ANOVA and different letters showed significant differences. Scale bars, 1 cm. Source data are provided as a Source Data file.

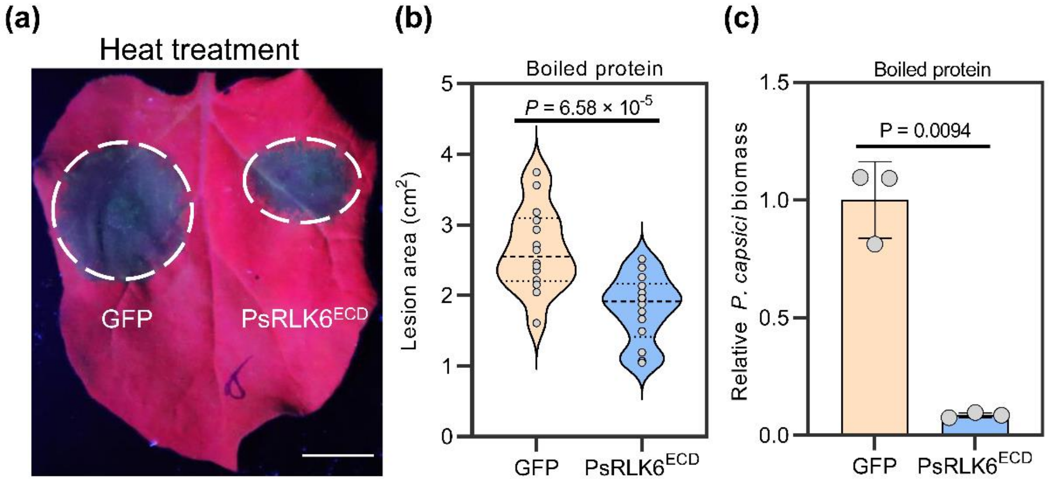

Supplementary Fig. 4. Disease resistance to *P. capsici* induced by boiled PsRLK6^{ECD} protein in *N. benthamiana* leaves. (a) Representative leaves showing the disease symptoms treated by boiled GFP *or* PsRLK6^{ECD} protein in *N. benthamiana* leaves upon infection with *P. capsici.* Scale bar, 1 cm. Lesion areas (b) and biomass of *P. capsici* infection (n=3 biologically independent samples) (c) were measured. The experiment was performed two times with similar results. In b, the data are shown as violin plots with individual data points plotted (n = 14 biologically independent samples). The centre line, edges and whiskers indicate the median, lower and upper quartiles and the minimum and maximum, respectively. Data were shown as mean ± SD. The statistical analyses were performed with two-tailed Student's *t*-test. Source data are provided as a Source Data file.

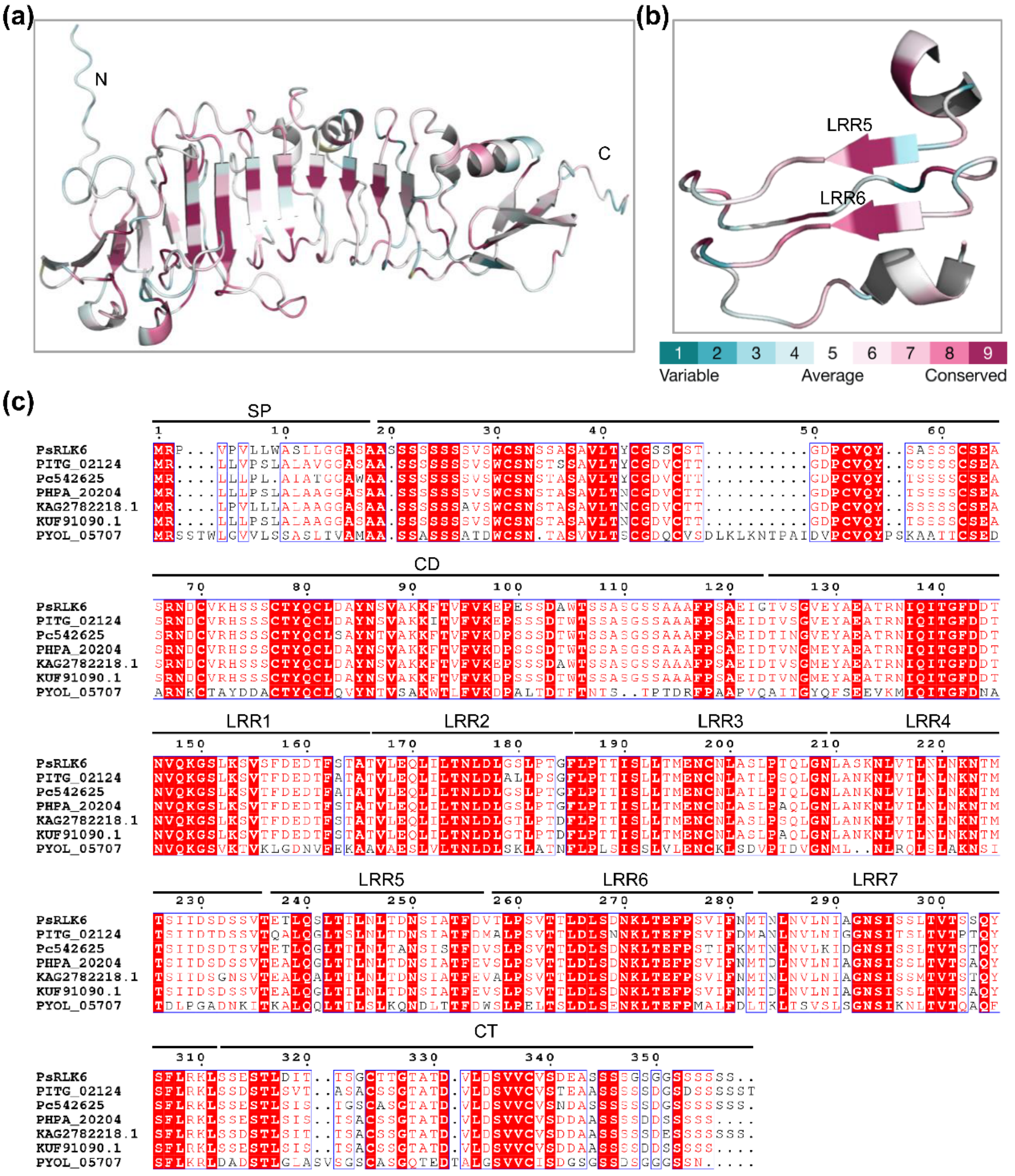

Supplementary Fig. 5. Analyses of conserved amino acid residues of PsRLK6^{ECD}. (a) Analyses of conserved amino cid residues of PsRLK6^{ECD} from obtained 54 orthologous gene were performed by the Consurf server. N represents N-terminal; C represents C-terminal. (b) Enlarge image of structure of LRR5-6. (c) Multiple sequence alignment of PsRLK6^{ECD} and its homologous sequences for assays from other oomycete species. PITG_021424 (*Phytophthora infestans*), Pc542625 (*P. capsici*), PHPA_20204 (*P. parasitica*), KAG2782218.1 (*P. cactorum*), KUF91090.1 (*P. nicotianae*) and PYOL_05707 (*Pythium oligandrum*) were selected for alignment analysis. Conserved residues are shaded on a red background. The signal peptide and LRR domains are indicated with black lines.

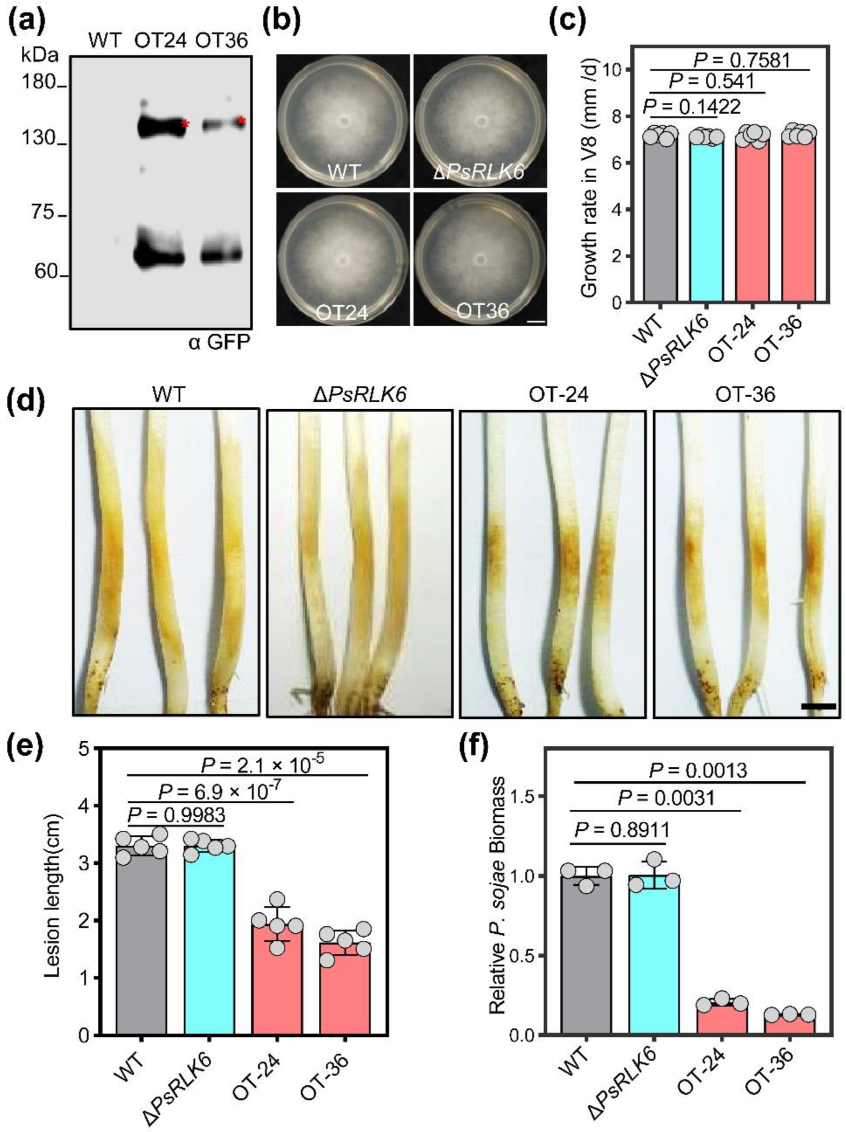

Supplementary Fig. 6. Overexpression of *PsRLK6* attenuates the virulence of *P. sojae*. (a) Immunoblot analysis of proteins isolated from *P. sojae* transformants overexpressing *PsRLK6-GFP* (OT24 and OT36) and from wild-type strain P6497. (b) Colony morphology of *P. sojae* wild-type and overexpression lines cultured on 10% V8 agar medium at 25°C in the dark for 7 days. Scale bar, 1 cm. (c) Growth rate of *P. sojae* WT and overexpression lines grown on V8 (n=6). (d) Visual symptoms 48 h after inoculation of etiolated soybean seedlings by *P. sojae* wild-type and overexpression lines. Representative photographs are shown (n=5). Scale bar, 1 cm. (e) Lesion length of WT and transgenic *P. sojae* strains infecting etiolated soybean hypocotyls (at 48 hpi) was measured. (f) Relative biomass of WT and overexpression *P. sojae* strains infecting etiolated soybean hypocotyls, as measured by qRT-PCR, and normalized to P6497 (n=3). The experiments were repeated three times with similar results. Data are shown as mean ± SD. *P* values were derived by two-tailed Student's t-test. Source data are provided as a Source Data file.

Supplementary Table 1. Primers used in this study.

Primers	Sequences 5'-3'	Used for
pSuper-PsRLK1^{ECD}-F	atacaccaaatcgactctagaATGGTGTCGCTGCGGGGC	cloning ECD of PsRLK1
pSuper-PsRLK1^{ECD}-R	gacgtcgtatgggtacccgggGAGTCCACCATGGTCGTCATCG	in *P. soaje*
pSuper-PsRLK2^{ECD}-F	atacaccaaatcgactctagaATGCCACTGCATCGCTGG	cloning ECD of PsRLK2
pSuper-PsRLK2^{ECD}-R	gacgtcgtatgggtacccgggGCGCCGCTGGTGCAGAGT	in *P. soaje*
pSuper-PsRLK3^{ECD}-F	atacaccaaatcgactctagaATGCAGGAAGGGCGCAAT	cloning ECD of PsRLK3
pSuper-PsRLK3^{ECD}-R	gacgtcgtatgggtacccgggAGAGTTTGCTTGCCACGCA	in *P. soaje*
pSuper-PsRLK4^{ECD}-F	atacaccaaatcgactctagaATGGCGGCGCGTCCATTA	cloning ECD of PsRLK4
pSuper-PsRLK4^{ECD}-R	gacgtcgtatgggtacccgggAGACGACGAAGATGACGATGTGC	in *P. soaje*
pSuper-PsRLK5^{ECD}-F	atacaccaaatcgactctagaATGAACTCACGCCACCGCG	cloning ECD of PsRLK5
pSuper-PsRLK5^{ECD}-R	gacgtcgtatgggtacccgggGTTGGTGTTATCAATCACGCTGC	in *P. soaje*
pSuper-PsRLK6^{ECD}-F	atacaccaaatcgactctagaATGCGCCCCGTACCGGTG	cloning ECD of PsRLK6
pSuper-PsRLK6^{ECD}-R	gacgtcgtatgggtacccgggGCTGCTACTAGATGAGGATCCTCC	in *P. soaje*
pSuper-PsRLK7^{ECD}-F	atacaccaaatcgactctagaATGCTGCGACTGGTGTCGG	cloning ECD of PsRLK7
pSuper-PsRLK7^{ECD}-R	gacgtcgtatgggtacccgggGTAGCTGTTGCTGCTGGACGA	in *P. soaje*
pSuper-PsRLK8^{ECD}-F	atacaccaaatcgactctagaATGCACGCCGTGCTCCGG	cloning ECD of PsRLK8
pSuper-PsRLK8^{ECD}-R	gacgtcgtatgggtacccgggGTTGCCGTTGCTGTTGCTG	in *P. soaje*
pSuper-PsRLK9^{ECD}-F	atacaccaaatcgactctagaATGCGCGGCGCTCGAACG	cloning ECD of PsRLK9
pSuper-PsRLK9^{ECD}-R	gacgtcgtatgggtacccgggCGGACTGCTGCTGCTCTCG	in *P. soaje*
pSuper-PsRLK10^{ECD}-F	atacaccaaatcgactctagaATGACAGGGGCGAGCAGAT	cloning ECD of
pSuper-PsRLK10^{ECD}-R	gacgtcgtatgggtacccgggGTTACTGAAGCCGCTGCTGC	PsRLK10 in *P. soaje*
pSuper-PsRLK11^{ECD}-F	atacaccaaatcgactctagaATGACGCAGTTGGCGGTTG	cloning ECD of
pSuper-PsRLK11^{ECD}-R	gacgtcgtatgggtacccgggTTCGGTGCACGTCCCTGA	PsRLK11 in *P. soaje*
pSuper-PsRLK12^{ECD}-F	atacaccaaatcgactctagaATGCTGCGCGCCTGGAGC	cloning ECD of
pSuper-PsRLK12^{ECD}-R	gacgtcgtatgggtacccgggGTGGCTGCTGCCGCCGCC	PsRLK12 in *P. soaje*
pSuper-PsRLK13^{ECD}-F	atacaccaaatcgactctagaATGCGCTGCCGTTGTGCT	cloning ECD of
pSuper-PsRLK13^{ECD}-R	gacgtcgtatgggtacccgggGTTGCTACTGCTGCCTCCACC	PsRLK13 in *P. soaje*
pSuper-PsRLK14^{ECD}-F	atacaccaaatcgactctagaATGGTCGCAGCCTACGCG	cloning ECD of
pSuper-PsRLK14^{ECD}-R	gacgtcgtatgggtacccgggCGGGCTCGTGCCACTCGA	PsRLK14 in *P. soaje*
pSuper-PsRLK15^{ECD}-F	atacaccaaatcgactctagaATGTCCTCGTCCTCTTCGTTGC	cloning ECD of
pSuper-PsRLK15^{ECD}-R	gacgtcgtatgggtacccgggTTTGTTGGAAGATGAATCCTCGT	PsRLK15 in *P. soaje*
pSuper-PsRLK16^{ECD}-F	atacaccaaatcgactctagaATGAGCGTTGACGCCAACC	cloning ECD of
pSuper-PsRLK16^{ECD}-R	gacgtcgtatgggtacccgggCGTCGATACAGAAGAACCCGC	PsRLK16 in *P. soaje*
pSuper-PsRLK17^{ECD}-F	atacaccaaatcgactctagaATGGCAGCAGGCGGTCGG	cloning ECD of
pSuper-PsRLK17^{ECD}-R	gacgtcgtatgggtacccgggCGTTGAAGAGGACGAGTCAGATG	PsRLK17 in *P. soaje*
pSuper-PsRLK18^{ECD}-F	atacaccaaatcgactctagaATGAGACGGACACGTCTGCAG	cloning ECD of
pSuper-PsRLK18^{ECD}-R	gacgtcgtatgggtacccgggCAACATCCAACCCGAAGACG	PsRLK18 in *P. soaje*
pSuper-PsRLK19^{ECD}-F	atacaccaaatcgactctagaATGAGGCTGTGGGCGTTGG	cloning ECD of
pSuper-PsRLK19^{ECD}-R	gacgtcgtatgggtacccgggGTTGGAGCTTGATCCTTCATTGA	PsRLK19 in *P. soaje*
pSuper-PsRLK20^{ECD}-F	atacaccaaatcgactctagaATGCACGCGTGGGCGCTG	cloning ECD of
pSuper-PsRLK20^{ECD}-R	gacgtcgtatgggtacccgggCGTCCTGTGGGATGAAGACG	PsRLK20 in *P. soaje*

Primers	Sequences 5'-3'	Used for
pSuper-PsRLK21^{ECD}-F	atacaccaaatcgactctagaATGACCTGGAGGTGGCTATTAGC	cloning ECD of
pSuper-PsRLK21^{ECD}-R	gacgtcgtatgggtacccgggCCCAGTCGCTATCCCTCCA	PsRLK21 in *P. soaje*
pSuper-PsRLK22^{ECD}-F	atacaccaaatcgactctagaATGACAAAGAAAGACTGGTCGAGG	cloning ECD of
pSuper-PsRLK22^{ECD}-R	gacgtcgtatgggtacccgggCGTCGATGTGTCGTGAACTGC	PsRLK22 in *P. soaje*
pSuper-PsRLK23^{ECD}-F	atacaccaaatcgactctagaATGGCACACGCGCAGAGC	cloning ECD of
pSuper-PsRLK23^{ECD}-R	gacgtcgtatgggtacccgggGTTGGAGCCGCCTGACGA	PsRLK23 in *P. soaje*
pSuper-PsRLK24^{ECD}-F	atacaccaaatcgactctagaATGAAGGCGGGCGCTGCA	cloning ECD of
pSuper-PsRLK24^{ECD}-R	atacaccaaatcgactctagaATGAAGGCGGGCGCTGCA	PsRLK24 in *P. soaje*
pBin-PsRLK6^{ECD}-F	gaacgatagggtacccccgggATGCGCCCCGTACCGGTG	cloning ECD of PsRLK6
pBin-PsRLK6^{ECD}-R	agtggatccgtcgaccccgggGCTGCTACTAGATGAGGATCCTCC	in *P. soaje*
pBin-PsRLK7^{ECD}-F	gaacgatagggtacccccgggATGCTGCGACTGGTGTCGG	cloning ECD of PsRLK7
pBin-PsRLK7^{ECD}-R	agtggatccgtcgaccccgggGTAGCTGTTGCTGCTGGACG	in *P. soaje*
pBin-PiRLK6^{ECD}-F	gaacgatagggtacccccgggATGCGTCTACTGGTGCCGTC	cloning PsRLK6^{ECD} homologous sequence
pBin-PiRLK6^{ECD}-R	agtggatccgtcgaccccgggGTCGTCCGCCAAGCTAACG	in *P. infestans*
pBin-PcRLK6^{ECD}-F	gaacgatagggtacccccgggATGCGTCTGCTGCTACCGTT	cloning PsRLK6^{ECD} homologous sequence
pBin-PcRLK6^{ECD}-R	agtggatccgtcgaccccgggGCTGGAAGATGAACTGCTTGATC	in *P. capsici*
pBin-PpRLK6^{ECD}-F	gaacgatagggtacccccgggATGCGTCTACTGCTGCCGTC	cloning PsRLK6^{ECD} homologous sequence
pBin-PpRLK6^{ECD}-R	agtggatccgtcgaccccgggGCTGGAAGACGAGCTGTCATCC	in *P. parasitica*
pBin-PnRLK6^{ECD}-F	gaacgatagggtacccccgggATGCGTCTACTGCTGCCGTC	cloning PsRLK6^{ECD} homologous sequence
pBin-PnRLK6^{ECD}-R	agtggatccgtcgaccccgggGTGCTGGAAGACGAGCTGTCA	in *P. nicotianae*
pBin-PcacRLK6^{ECD}-F	gaacgatagggtacccccgggATGCGTCTACCGGTATTGCTG	cloning PsRLK6^{ECD} homologous sequence
pBin-PcacRLK6^{ECD}-R	agtggatccgtcgaccccgggGCTGGAAGACGAGCTGCTTG	in *P. cactorum*
pBin-PyolRLK6^{ECD}-F	gaacgatagggtacccccgggATGGCGGCCACATCTCGTTC	cloning PsRLK6^{ECD} homologous sequence
pBin-PyolRLK6^{ECD}-R	agtggatccgtcgaccccgggGTTGCTCGAGCCACCACCG	in *Py. oligandrum*
pPICZaA-PsRLK6^{ECD}-F	gagaggctgaagcttacgtgATGGCCTCGTCCTCCTCGTCG	cloning ECD of PsRLK6
pPICZaA-PsRLK6^{ECD}-R	cagtggtggtggtggtggtgGCTGCTACTAGATGAGGATCCTCC	in *P. soaje*
pPICZaA-PsRLK7^{ECD}-F	gagaggctgaagcttacgtgATGGCCTCCTCCTCCTCCTCTAGC	cloning ECD of PsRLK7
pPICZaA-PsRLK7^{ECD}-R	cagtggtggtggtggtggtgGTAGCTGTTGCTGCTGGACG	in *P. soaje*
pBin-PsRLK6^{ECD}-M1-F	gaacgatagggtacccccgggATGCGCCCCGTACCGGTG	
pBin-PsRLK6^{ECD}-M1-R	agcgtttcagtgaccgaggaGTTGAAGATGACTGAGGGGA	
pBin-PsRLK6^{ECD}-M2-F	gaacgatagggtacccccgggATGCGCCCCGTACCGGTG	cloning truncation of
pBin-PsRLK6^{ECD}-M2-R	agcgtttcagtgaccgaggaATCAAAAGTGGCGATCGAGT	PsRLK6^{ECD} in *P. soaje*
pBin-PsRLK6^{ECD}-M3-F1	gaacgatagggtacccccgggATGCGCCCCGTACCGGTG	for ROS accumulation
pBin-PsRLK6^{ECD}-M3-R1	tactcgacgcccgagaccgtCGCCGAGGCGCCGCCCAG	and inoculation assays
pBin-PsRLK6^{ECD}-M3-F2	ACGGTCTCGGGCGTCGAG	
pBin-PsRLK6^{ECD}-M3-R2	agtggatccgtcgaccccgggGCTGCTACTAGATGAGGATCCTCC	

pBin-PsRLK6^{ECD}-M4-F1	gaacgatagggtacccccgggATGCGCCCCGTACCGGTG	
pBin-PsRLK6^{ECD}-M4-R1	tcgtcaagctctgcagcgtCGCCGAGGCGCCGCCCAG	
pBin-PsRLK6^{ECD}-M4-F2	gACGCTGCAGAGCTTGACGA	
pBin-PsRLK6^{ECD}-M4-R2	agtggatccgtcgaccccgggGCTGCTACTAGATGAGGATCCTCC	
pBin-PsRLK6^{ECD}-M5-F1	gaacgatagggtacccccgggATGCGCCCCGTACCGGTG	
pBin-PsRLK6^{ECD}-M5-R1	tggtaacggatggaagcgtCGCCGAGGCGCCGCCCAG	
pBin-PsRLK6^{ECD}-M5-F2	gACGCTTCCATCCGTTACCACA	
pBin-PsRLK6^{ECD}-M5-R2	agtggatccgtcgaccccgggGCTGCTACTAGATGAGGATCCTCC	
pBin-PsRLK6^{ECD}-M6-F1	gaacgatagggtacccccgggATGCGCCCCGTACCGGTG	
pBin-PsRLK6^{ECD}-M6-R1	tcgtcaagctctgcagcgtCGCCGAGGCGCCGCCCAG	
pBin-PsRLK6^{ECD}-M6-F2	gACGCTGCAGAGCTTGACGA	
pBin-PsRLK6^{ECD}-M6-R2	agtggatccgtcgaccccgggTTTCCTCAAAAAGCTGTACTGTGAC	
pBin-PsRLK6^{ECD}-M7-F1	gaacgatagggtacccccgggATGCGCCCCGTACCGGTG	
pBin-PsRLK6^{ECD}-M7-R1	tcgtcaagctctgcagcgtCGCCGAGGCGCCGCCCAG	
pBin-PsRLK6^{ECD}-M7-F2	gACGCTGCAGAGCTTGACGA	
pBin-PsRLK6^{ECD}-M7-R2	agtggatccgtcgaccccgggGCTGCTACTAGATGAGGATCCTCC	
pBin-PsRLK6^{ECD}-M8-F1	agtggatccgtcgaccccgggGTTGAAGATGACTGAGGGGAACTC	
pBin-PsRLK6^{ECD}-M8-R1	tggtaacggatggaagcgtCGCCGAGGCGCCGCCCAG	
pBin-PsRLK6^{ECD}-M8-F2	gACGCTTCCATCCGTTACCACA	
pBin-PsRLK6^{ECD}-M8-R2	agtggatccgtcgaccccgggGTTGAAGATGACTGAGGGGAACTC	
NbEF1α-RT-F	GTATGCCTGGGTGCTTGAC	qPCR
NbEF1α-RT-R	ACAGGGACAGTTCCAATACCA	
NbPR1a-RT-F	CCGCCTTCCCTCAACTCAAC	qPCR
NbPR1a-RT-R	GCACAACCAAGACGTACTGAG	
NbCYP71D20-RT-F	CCGCACCATGTCCTTAGAG	qPCR
NbCYP71D20-RT-R	CTTGCCCCTTGAGTACTTGC	
GmActin 4-RT-*F*	GATCTACCATGTTCCCAAGT	qPCR
GmActin 4-RT-*R*	ATAGAGCCACCAATCCAGAC	
GmPR1-RT-F	GGCCAATACGGGGAGAATCT	qPCR
GmPR1-RT-R	TCCAAACAACCTGAGTGTAATGC	
AtPR1-RT-F	TGGTCACTACACTCAAGTTGTT	qPCR
AtPR1-RT-R	GCTTCTCGTTCACATAATTCCC	
ToPR1-RT-F	TACGCTACCAACCAATGTG	qPCR
ToPR1-RT-R	TCCAGTTGCCTACAGGATC	
NbBAK1-RT-F	GAGGTGGGAGGAATGGCAAA	qPCR
NbBAK1-RT-R	TTGGCCCCGACAATTCATCT	
pTOR-PsRLK6-GFP-F	ctcgaggtcgacggtatcgat ATGCGCCCCGTACCGGTGCTC	For construct PsRLK6 overexpression transformant
pTOR-PsRLK6-GFP-R	ggtgaattcgatatcatcgatATCGGCCGCCAGACTGACTG	
