# Supplementary material for: A Phytophthora receptor-like kinase regulates oospore development and can activate pattern-triggered plant immunity
Source: Nat Commun. 2023 Jul 31;14:4593. doi: 10.1038/s41467-023-40171-7 (PMC10390575; doi:10.1038/s41467-023-40171-7)
Supplement: Supplementary file 3 — Description of suppl. data_new [file 41467_2023_40171_MOESM3_ESM.docx]

**Description of Additional Supplementary Files**

File name: Supplementary Data 1
Description: List of all PsRLK6 homologs in selected species.

File name: Supplementary Data 2

Description: The list of single copy genes used in the phylogenetic analysis.

File name: Supplementary Data 3

Description: The output file of structure of PsRLK6^ECD^ predicted by ColabFold. The file includes:

1. PDB formatted structures sorted by avg. pLDDT and complexes are sorted by pTMscore.
2. Plots of the model quality.
3. Plots of the MSA (Multiple Sequence Alignment) coverage.
4. Parameter log file.
5. A3M formatted input MSA.
6. A predicted_aligned_error_v1.json using [AlphaFold-DB's format](https://alphafold.ebi.ac.uk/faq#faq-7) and a scores.json for each model which contains an array (list of lists) for PAE, a list with the average pLDDT and the pTMscore.
7. BibTeX file with citations for all used tools and databases.

A more detailed description can be found on the website <https://colab.research.google.com/github/sokrypton/ColabFold/blob/main/AlphaFold2.ipynb>
