# Supplementary material for: A Phytophthora receptor-like kinase regulates oospore development and can activate pattern-triggered plant immunity
Source: Nat Commun. 2023 Jul 31;14:4593. doi: 10.1038/s41467-023-40171-7 (PMC10390575; doi:10.1038/s41467-023-40171-7)
Supplement: Supplementary file 6 — Supplementary data 3_new [file 41467_2023_40171_MOESM6_ESM.zip › 40171/Readme link for Suppl. Data 3.docx]

The github link to navigate the content of the Suppl. Data 3 folders. <https://colab.research.google.com/github/sokrypton/ColabFold/blob/main/AlphaFold2.ipynb>
