# Supplementary material for: A Phytophthora receptor-like kinase regulates oospore development and can activate pattern-triggered plant immunity
Source: Nat Commun. 2023 Jul 31;14:4593. doi: 10.1038/s41467-023-40171-7 (PMC10390575; doi:10.1038/s41467-023-40171-7)
Supplement: Supplementary file 7 — Reporting Summary [file 41467_2023_40171_MOESM7_ESM.pdf]

nature portfolio

Corresponding author(s): Daolong Dou; Zhiyuan Yin

Last updated by author(s): Jun 19, 2023

Reporting Summary

Nature Portfolio wishes to improve the reproducibility of the work that we publish. This form provides structure for consistency and transparency in reporting. For further information on Nature Portfolio policies, see our Editorial Policies and the Editorial Policy Checklist.

Statistics

For all statistical analyses, confirm that the following items are present in the figure legend, table legend, main text, or Methods section.

n/a	Confirmed	
☐	☒	The exact sample size (*n*) for each experimental group/condition, given as a discrete number and unit of measurement
☐	☒	A statement on whether measurements were taken from distinct samples or whether the same sample was measured repeatedly
☐	☒	The statistical test(s) used AND whether they are one- or two-sided<br>*Only common tests should be described solely by name; describe more complex techniques in the Methods section.*
☒	☐	A description of all covariates tested
☐	☒	A description of any assumptions or corrections, such as tests of normality and adjustment for multiple comparisons
☐	☒	A full description of the statistical parameters including central tendency (e.g. means) or other basic estimates (e.g. regression coefficient) AND variation (e.g. standard deviation) or associated estimates of uncertainty (e.g. confidence intervals)
☐	☒	For null hypothesis testing, the test statistic (e.g. *F*, *t*, *r*) with confidence intervals, effect sizes, degrees of freedom and *P* value noted<br>*Give P values as exact values whenever suitable.*
☒	☐	For Bayesian analysis, information on the choice of priors and Markov chain Monte Carlo settings
☒	☐	For hierarchical and complex designs, identification of the appropriate level for tests and full reporting of outcomes
☒	☐	Estimates of effect sizes (e.g. Cohen's *d*, Pearson's *r*), indicating how they were calculated

Our web collection on statistics for biologists contains articles on many of the points above.

Software and code

Policy information about availability of computer code

Data collection	The protein structure predicted by ColabFold 1.5.2 was stated in the method section.
Data analysis	GraphPad Prism 9, Pymol 2.4.0, ImageJ v1.52, MEGA 11, BLASTP (v2.5.0), TribeMCL v14.137, MUSCLE v3.8.31.

For manuscripts utilizing custom algorithms or software that are central to the research but not yet described in published literature, software must be made available to editors and reviewers. We strongly encourage code deposition in a community repository (e.g. GitHub). See the Nature Portfolio guidelines for submitting code & software for further information.

Data

Policy information about availability of data

All manuscripts must include a data availability statement. This statement should provide the following information, where applicable:

- Accession codes, unique identifiers, or web links for publicly available datasets
- A description of any restrictions on data availability
- For clinical datasets or third party data, please ensure that the statement adheres to our policy

All data are available within the article and supplementary Files.The proteomes of different organisms used in this study can be obtained from National Center for Biotechnology Information (NCBI) and JGI databases with following accession numbers: Phytophthora sojae (GCA_000149755.2), P. infestans (GCA_000142945.1), P. nicotianae (GCA_001483015.1), P. aleatoria (GCA_018873745.1), P. pseudosyringae (GCA_019155715.1), P. fragariae (GCA_009733025.1), P. rubi (GCA_009732945.1), P. capsici (GCA_000325885.1), P. idaei (GCA_016880175.1), P. cactorum (GCA_003287315.1), P. kernoviae (GCA_001712705.2), P. parasitica (GCA_000509465.1), Hyaloperonospora rabidopsis (GCA_000173235.2), Bremia lactucae (GCA_004359215.2), Plasmopara halstedii (GCA_900000015.1), Pythium

ultimum (GCA_000143045.1), Pythium oligandrum (GCA_005966545.1), Pythium aphanidermatum (GCA_000387445.2), Globisporangium splendens (GCA_006386115.1), Albugo laibachii (GCA_902706625.1), Albugo candida (GCA_001078535.1), Aphanomyces invadans (GCA_000520115.1), Aphanomyces astaci (GCA_003546625.1), Saprolegnia diclina (GCA_000281045.1), Saprolegnia parasitica (GCA_000151545.2), Thalassiosira pseudonana (GCA_000149405.2), Botrytis cinerea (GCA_000143535.4), Magnaporthe oryzae (GCA_000002495.2), Sclerotinia sclerotiorum (GCA_001857865.1), Pseudomonas syringae (GCA_002905815.2), Xanthomonas oryzae (GCA_008370835.2), Arabidopsis thaliana (GCA_001651475.1), and Glycine max (GCA_000004515.5). Source data are provided with this paper.

Human research participants

Policy information about studies involving human research participants and Sex and Gender in Research.

Reporting on sex and gender	Not applicable.
Population characteristics	Not applicable.
Recruitment	Not applicable.
Ethics oversight	Not applicable.

Note that full information on the approval of the study protocol must also be provided in the manuscript.

Field-specific reporting

Please select the one below that is the best fit for your research. If you are not sure, read the appropriate sections before making your selection.

[x] Life sciences   [ ] Behavioural & social sciences   [ ] Ecological, evolutionary & environmental sciences

For a reference copy of the document with all sections, see nature.com/documents/nr-reporting-summary-flat.pdf

Life sciences study design

All studies must disclose on these points even when the disclosure is negative.

Sample size	Sample size was determined based on previous publications on similar experimental trials. At least three biological replicates were performed in all experiments.
Data exclusions	No data that pass quality control were excluded from analysis. All original data is included in the Source Data.
Replication	Each experiment was successfully repeated independently at least three times. Results were reproducible in all repeats with the same trend.
Randomization	For each independent experiment, Nicotiana benthamiana leaves were selected with similar sizes and at the same position in each plant to minimize the side effects caused by plant growth. These plants were randomly assigned to the treatment and control groups with no formal randomization techniques.
Blinding	The infection, DAB staining and western blot assays were recorded in a blind way. Experiment results are not subjective.

Reporting for specific materials, systems and methods

We require information from authors about some types of materials, experimental systems and methods used in many studies. Here, indicate whether each material, system or method listed is relevant to your study. If you are not sure if a list item applies to your research, read the appropriate section before selecting a response.

Materials & experimental systems

n/a	Involved in the study	
[ ]	[x]	Antibodies
[x]	[ ]	Eukaryotic cell lines
[x]	[ ]	Palaeontology and archaeology
[x]	[ ]	Animals and other organisms
[x]	[ ]	Clinical data
[x]	[ ]	Dual use research of concern

Methods

n/a	Involved in the study	
[x]	[ ]	ChIP-seq
[x]	[ ]	Flow cytometry
[x]	[ ]	MRI-based neuroimaging

Antibodies

Antibodies used	Anti-HA (Catalog number: 12013819001; Sigma-Aldrich;1:5000)<br>Anti-GFP (Catalog number: M2004L; Abmart; 1:5000)<br>Anti-phospho-p44/42 MAPK (Erk1/2) antibody (Catalog number:#4370s; Cell Signaling, 1:1000)
Validation	Anti-HA antibody manufacturer's website: https://www.sigmaaldrich.cn/CN/zh/product/roche/12013819001<br>Anti-GFP antibody manufacturer's website: http://www.ab-mart.com.cn/page.aspx?node=%2059%20&id=%20971<br>Anti-phospho-p44/42 MAPK (Erk1/2) antibody manufacturer's website:https://www.cellsignal.cn/products/primary-antibodies/phospho-p44-42-mapk-erk1-2-thr202-tyr204-d13-14-4e-xp-rabbit-mab/4370?site-search-type=Products&N=4294956287&Ntt=anti-phospho-p44%2F42+mapk+&fromPage=plp
